# Biochemical investigation of the tryptophan biosynthetic enzyme anthranilate phosphoribosyltransferase in plants

**DOI:** 10.1016/j.jbc.2023.105197

**Published:** 2023-08-31

**Authors:** Miriam Li, Hisham Tadfie, Cameron G. Darnell, Cynthia K. Holland

**Affiliations:** Department of Biology, Williams College, Williamstown, Massachusetts, USA

**Keywords:** tryptophan, enzyme kinetics, substrate specificity, plant biochemistry, anthranilate metabolism, metabolic regulation

## Abstract

While mammals require the essential amino acid tryptophan (Trp) in their diet, plants and microorganisms synthesize Trp *de novo*. The five-step Trp pathway starts with the shikimate pathway product, chorismate. Chorismate is converted to the aromatic compound anthranilate, which is then conjugated to a phosphoribosyl sugar in the second step by anthranilate phosphoribosyltransferase (PAT1). As a single-copy gene in plants, all fixed carbon flux to indole and Trp for protein synthesis, specialized metabolism, and auxin hormone biosynthesis proceeds through PAT1. While bacterial PAT1s have been studied extensively, plant PAT1s have escaped biochemical characterization. Using a structure model, we identified putative active site residues that were variable across plants and kinetically characterized six PAT1s (*Arabidopsis thaliana* (thale cress), *Citrus sinensis* (sweet orange), *Pistacia vera* (pistachio), *Juglans regia* (English walnut), *Selaginella moellendorffii* (spike moss), and *Physcomitrium patens* (spreading earth-moss)). We probed the catalytic efficiency, substrate promiscuity, and regulation of these six enzymes and found that the *C. sinensis* PAT1 is highly specific for its cognate substrate, anthranilate. Investigations of site-directed mutants of the *A. thaliana* PAT1 uncovered an active site residue that contributes to promiscuity. While Trp inhibits bacterial PAT1 enzymes, the six plant PAT1s that we tested were not modulated by Trp. Instead, the *P. patens* PAT1 was inhibited by tyrosine, and the *S. moellendorffii* PAT1 was inhibited by phenylalanine. This structure-informed biochemical examination identified variations in activity, efficiency, specificity, and enzyme-level regulation across PAT1s from evolutionarily diverse plants.

Plants synthesize an abundance of metabolites to aid in growth and development, defense against pathogens and herbivores, the mitigation of environmental stresses, and reproduction. Many plant secondary and specialized metabolites are synthesized from amino acids or their metabolic intermediates. In plants, the aromatic amino acid tryptophan (Trp) is essential for protein synthesis and is a precursor to auxin growth hormones, alkaloids, niacin (vitamin B_3_), and anti-herbivory metabolites like indole glucosinolates (1, 2, 3, 4, 5). While plants, fungi, and bacteria have retained the enzymes necessary to synthesize Trp *de novo*, humans require the essential amino acid Trp from their diet (1, 2). Although Trp is essential for protein synthesis, specialized metabolism, and human health, much of what is known about aromatic amino acid biosynthesis and regulation has been inferred from microbial investigations of these pathways and has largely escaped biochemical investigation in plants.

The five-step, plastid-localized Trp pathway starts with chorismate, the product of the shikimate pathway, which is converted to anthranilate (2-aminobenzoate) and pyruvate by anthranilate synthase (AS) (Fig. 1) (6, 7, 8, 9, 10, 11). In the second step of the pathway, phosphoribosyl anthranilate transferase (PAT1) transfers a phosphoribosyl sugar onto anthranilate, forming 5-phosphoribosylanthranilate (12, 13, 14). The *PAT1*, or *TRP1*, gene was the first Trp pathway enzyme discovered in plants (13). Because *PAT1* is a single-copy gene in Arabidopsis, *trp1-1* plants are Trp auxotrophs. The *trp1* mutants also exhibit blue fluorescence under UV light due to the accumulation of anthranilate β-glucoside (15).

**Figure 1:**
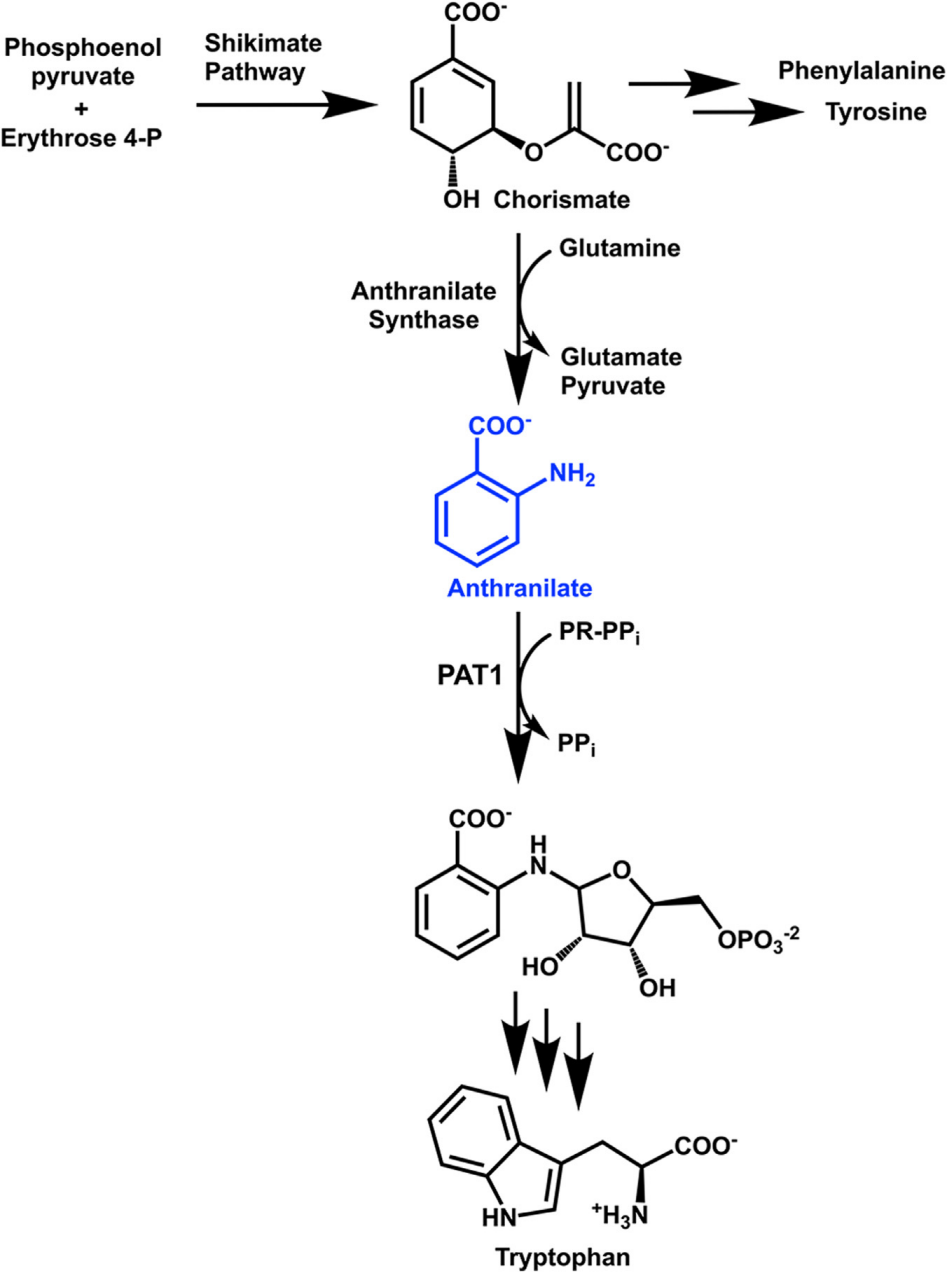
**Anthranilate phosphoribosyltransferase (PAT1) catalyzes the second step in Trp biosynthesis**. Phosphoenol pyruvate from glycolysis and erythrose 4-phosphate from the Calvin-Benson cycle are shuttled through the shikimate pathway to generate the precursor of aromatic amino acid biosynthesis, chorismate. Chorismate is then converted to anthranilate, which is phosphoribosylated by PAT1 in the second step of the five-step Trp pathway.

In aromatic amino acid biosynthesis, the first enzyme in the pathway is typically regulated by allosteric effectors (2). In Tyr and Phe biosynthesis, chorismate mutase 1 (CM1) in *Arabidopsis thaliana* is allosterically inhibited by Phe and Tyr and activated by Trp, while CM3 is activated by all three aromatic amino acids, Cys, and His (16). In *Physcomitrium patens*, both CM1 and CM2 are allosterically activated by Trp and inactivated by Tyr and Phe, while His is an activator only for CM1 (17). The *Selaginella moellendorffii* CM is allosterically activated by Trp but is not allosterically regulated by Tyr, Phe, or His. In Trp biosynthesis, AS is feedback regulated through allosteric inhibition by the final pathway product, Trp, although ASαs that are feedback-insensitive to Trp have been identified in rice, common rue, and tobacco (6, 18, 19). While these enzymes at the chorismate branch point are modulated by amino acid effectors, steps within the pathway are regulated by competitive inhibition. For example, arogenate dehydrogenase in Tyr biosynthesis is inhibited by Tyr and prephenate aminotransferase at the branchpoint of Tyr and Phe biosynthesis is inhibited by Cys (20, 21). While feedback inhibition by Trp has been identified in a bacterial PAT, whether or not plant PAT1s are regulated and the molecular determinants of enzyme-level regulation are unknown, and our understanding of the regulation of Trp biosynthetic enzymes remains incomplete (22).

Several examples exist where plants siphon away intermediates in the Trp biosynthesis pathway to use as starting material for building specialized metabolites. In maize, indole-3-glycerol phosphate is drawn from Trp biosynthesis to produce benzoxazinoids, which are used for defense against insect herbivores (4, 23). Anthranilate is methylated in plants such as maize (*Zea mays*), fox grapes (*Vitis labrusca*), and strawberries (*Fragaria* spp.) to generate *O*-methyl anthranilate, which serves to both attract pollinators and repel herbivores (24, 25, 26, 27). *O*-Methyl anthranilate is responsible for the recognizable smell of grapes and is used commercially as a grape flavoring agent in foods, beverages, and pharmaceuticals and as a bird deterrent on golf courses (26). In the Citrus family (Rutaceae), *N*-methyl anthranilate serves as a precursor in acridone alkaloid biosynthesis (28). Acridone, acridine, and their derivatives are toxic to human cell lines and have broad biological activity against bacteria, viruses, and parasites, suggesting that they serve as a chemical defense mechanism in plants (29). As a branch point in primary and specialized metabolism, we hypothesized that plants may modulate anthranilate flux to Trp *via* PAT1 regulation.

Despite the fundamental role of anthranilate as a precursor to Trp and defensive molecules, PAT1 remains to be biochemically characterized outside of microorganisms. To understand PAT1 enzyme activity, specificity, and regulation, we combined structural comparisons of active site residues with functional assays. Our functional analysis included PAT1s from *A. thaliana* (thale cress), *Citrus sinensis* (sweet orange), *Pistacia vera* (pistachio), *Juglans regia* (English walnut), *S. moellendorffii* (spike moss), and *P. patens* (spreading earth-moss). The PAT1 proteins from these species were selected because the active sites of these enzymes were the most variable, which enabled us to identify residues that may contribute to variations in activity and regulation. While there were many conserved residues in the active sites across these enzymes, they displayed variability in catalytic efficiencies, substrate inhibition, and substrate promiscuity. Key residues in the *A. thaliana* PAT1 were investigated for their role in substrate specificity, which identified a Ser that is key for limiting non-cognate reactions. We tested four amino acids (Trp, Tyr, Phe, and His) as effectors of each of the plant PAT1s and found that Phe is an inhibitor of the *S. moellendorffii* PAT1, Tyr is an inhibitor of the *P. patens* PAT1, and the other four PAT1s were insensitive to amino acid regulation. Our results indicate that plant PAT1s have evolved differential activities, specificities, and modulators based on their metabolic landscapes and selective pressures for balancing Trp biosynthesis with anthranilate-derived specialized metabolism.

## Results

### Structural comparisons of active site residues across plant PAT1s

**Figure 2:**
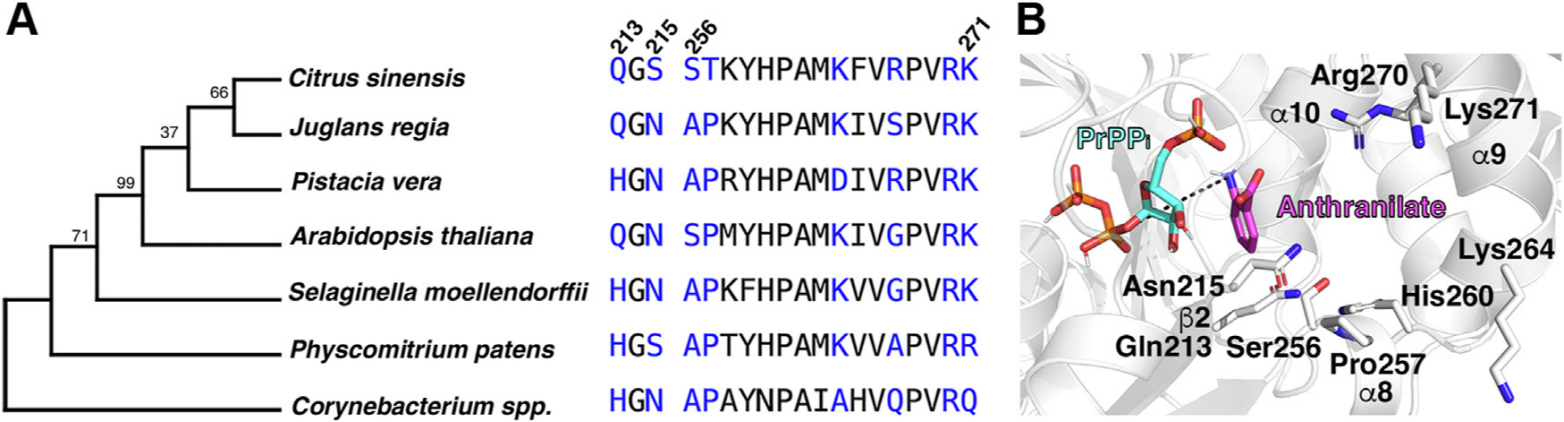
**Phylogeny-guided structural analysis of the PAT1 active site.***A*, a dendrogram of PAT1 proteins from plants and a microorganism (*Corynebacterium spp*.) shows the evolutionary relationship among selected PAT1 enzymes. Active site residues for each PAT1 are shown on the *left*, and residues in *blue* were within 5 Å of a docking conformation of anthranilate (panel *B*, *magenta*). Amino acid numbering is based on the position in the *A. thaliana* PAT1 protein. *B*, most identified active site residues are located on the β2 sheet and the α8 and α9 helices. The *black dashed line* between the amine of anthranilate and PrPPi (phosphoribosyl-pyrophosphate) shows the point at which the transfer reaction occurs.

To determine the extent of variation across plant PAT1s, we began by visualizing the differences between computed structure models of a PAT1 from *A. thaliana*, AtPAT1. The location of the active site was confirmed by overlaying an anthranilate-bound archaeal PAT1 structure (PDB: 2GVQ) with the AtPAT1 model (30). The sugar donor, 5-phospho-D-ribose 1-diphosphate (PR-PP_i_), was docked into the active site, and the PR-PP_i_-bound structure was used for molecular docking of anthranilate (Fig. 2). X-ray crystal structures have shown that the active sites of microbial PAT1s sit at the hinge region between the N-terminal α-helical domain and a C-terminal α/β domain and contain multiple sites for anthranilate binding, a so-called anthranilate channel, with the S1 site being closest to the PR-PP_i_ to facilitate catalysis and the distal S2 site allowing for substrate capture (31, 32).

These anthranilate-bound models allowed us to identify residues that shape the molecular architecture of the active site. Most active site residues fell in two regions on the β2 sheet (213–215) and helix α8 (256–259) and α9 (262–271). There are only three residues within 5 Å of docked anthranilate that are variable between *Arabidopsis* and *Citrus*: Pro257 is a Thr in the CsPAT1, Asn215 is a Ser in CsPAT1, and Gly267 is an Arg in CsPAT1. Several charged Lys and Arg residues on helix α9 are highly conserved and are likely involved in electrostatic interactions between the carboxylate of anthranilate (Fig. 2).

**Figure 3:**
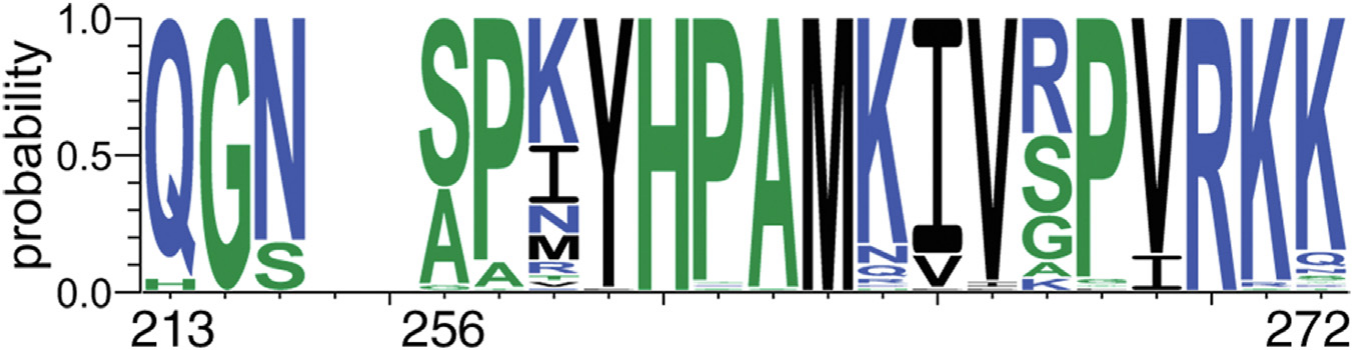
**Comparisons of 82 plant PAT1 active site residues reveal highly conserved and variable residues.** Amino acid numbering is based on the position in the *A. thaliana* PAT1 protein. Figure was designed using WebLogo3.

To dig deeper into the variability of PAT1s across plants, we identified an additional 76 PAT1 orthologs across Viridiplantae for active site comparisons (File S1). While some residues were highly conserved (*e.g.*, Gly214, Tyr259, His260, Met263, and Arg270), others were more variable (*e.g.*, positions 256, 258, and 267) (Fig. 3). Instead of testing the role of each residue individually, we reasoned that assaying diverse plant PAT1s would allow us to gain a broader understanding of how these active site residue combinations contribute to the activity, specificity, and regulation of this key Trp biosynthetic enzyme across the plant kingdom. We selected six PAT1s with varied active sites for functional characterization: *A. thaliana* (thale cress), *C. sinensis* (sweet orange), *P. vera* (pistachio), *J. regia* (English walnut), *S. moellendorffii* (spike moss), and *P. patens* (spreading earth-moss).

### Functional characterization of PAT1s

**Figure 4:**
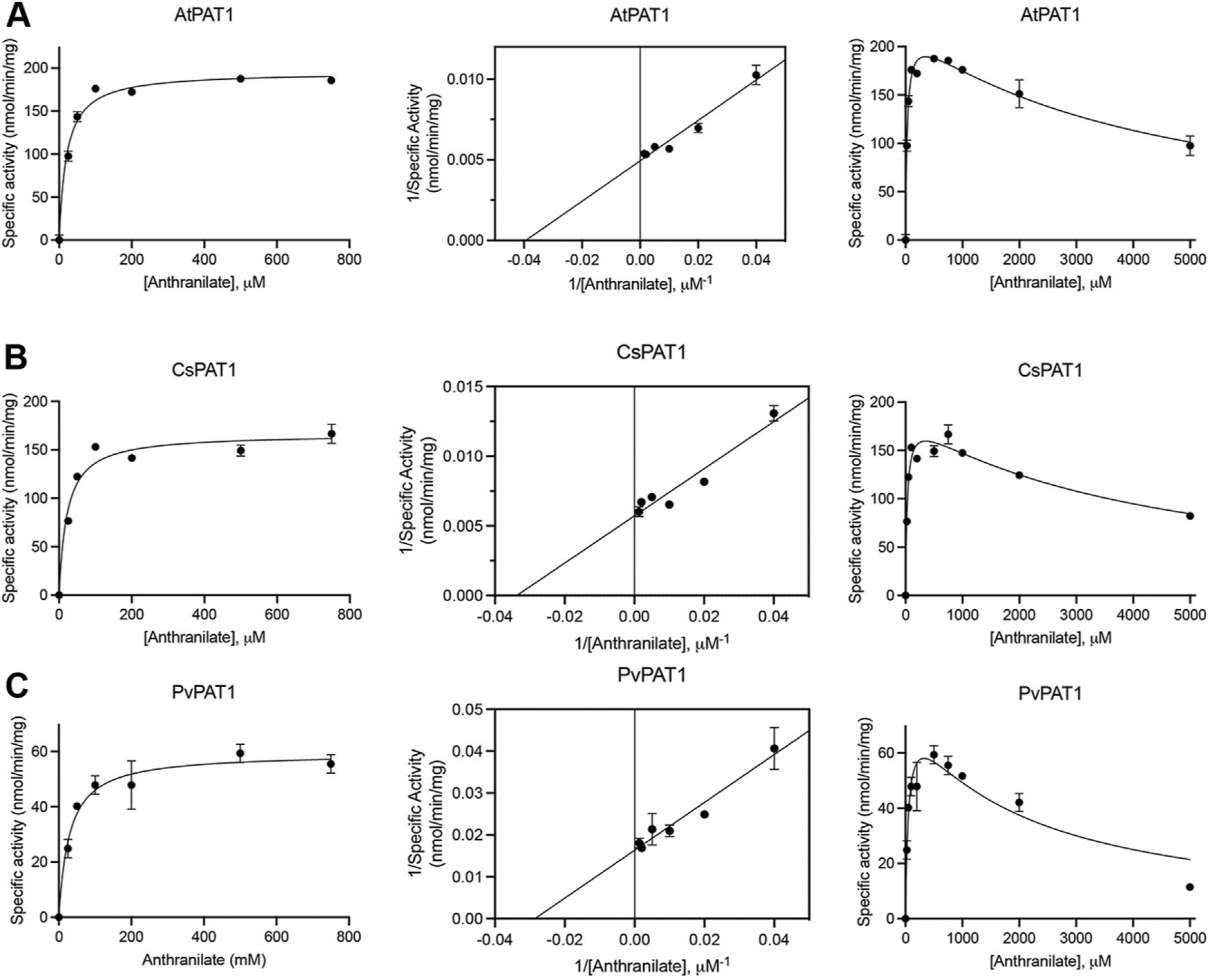
**Kinetic plots of three representative PAT1s.** Plots are shown for *Arabidopsis thaliana* (AtPAT1; *A*), *Citrus sinensis* (CsPAT1; *B*), and *Pistacia vera* (PvPAT1; *C*) with varied anthranilate. Data are represented as Michaelis-Menten (*left*), Lineweaver-Burke (*center*), and substrate-inhibition (*right*) plots for varied anthranilate concentrations. Error bars represent standard deviations, where bars are not visible, the standard deviation is too small to visualize. For all points, n = 3.

PAT1 uses anthranilate and PR-PP_i_ as substrates in a transferase reaction to form 5-phosphoribosylanthranilate (Fig. 1) (14). To investigate the kinetic differences between the plant PAT1s, the codon-optimized genes were cloned into the IPTG-inducible pET28a protein expression vector for heterologous expression in *E. coli*. Yields from Ni-NTA affinity purification ranged from 2 to 10 mg/ml. Purified enzymes were assayed using a continuous coupling enzyme reagent that couples PP_i_ release to NADH oxidation at 340 nm. Using this assay, we determined the steady-state kinetics for PAT1s by varying the concentration of anthranilate (Table 1 and Fig. 4).

**Table 1:** Steady state kinetics data for PAT1 enzymes with varied anthranilate

PAT1 protein	K_M_ (μM)	k_cat_ (min^−1^)	k_cat_/K_M_ (M^−1^s^−1^)	K_i_ (mM)
*Arabidopsis thaliana*	20.6 ± 2.0	7.9 ± 0.1	6360	4.3 ± 0.4
*Citrus sinensis*	22.0 ± 3.3	7.0 ± 0.2	5320	4.1 ± 0.6
*Juglans regia*	38.7 ± 3.5	3.3 ± 0.1	1420	>5
*Physcomitrium patens*	31.8 ± 4.4	3.4 ± 0.1	1790	>5
*Pistacia vera*	32.1 ± 4.7	2.4 ± 0.1	1240	1.8 ± 0.4
*Selaginella moellendorffii*	189 ± 35	5.1 ± 0.3	450	3.6 ± 0.8

K_M_ apparent, k_cat_, and k_cat_/K_M_ values reported for anthranilate concentrations between 0 and 0.75 mM. Values shown are the mean ± standard error for n = 3.

Across the various PAT1s, the K_M_ values mostly range from 20.6 to 38.7 μM when anthranilate was varied, with the exception of the *S. moellendorffii* PAT1 which has a notably high K_M_ (189 μM) (Table 1). The *A. thaliana* and *C. sinensis* enzymes had the highest k_cat_ (7.9 and 7.0 min^−1^, respectively) and catalytic efficiencies (6360 and 5320 M^−1^s^−1^, respectively) of the PAT1s that we tested. For *S. moellendorffii*, the high K_M_ value coupled with a moderate turnover rate (5.1 min^−1^) translated to a low catalytic efficiency (450 M^−1^s^−1^), which was the lowest of any of the PAT1s that we assayed. *P. vera* had the lowest turnover (2.4 min^−1^) and a catalytic efficiency of 1240 M^−1^s^−1^, which is 2.75-fold higher than the *S. moellendorffii* PAT1 yet 5-fold lower than the *A. thaliana* PAT1.

Substrate inhibition is a common deviation from Michaelis-Menten kinetics and plays a critical role in regulating metabolic pathways (33). Substrate inhibition with anthranilate has also been reported for *Mycobacterium tuberculosis* PAT1 (K_i_ value of 45 μM) and *Thermococcus kodakarensis* PAT1 (K_i_ value > 4 μM) (31, 34). While all the PAT1 enzymes also exhibit substrate inhibition kinetics, there was variability in the inhibitory constant (K_i_) values, and we were unable to calculate K_i_ for *J. regia* and *P. patens*. The K_i_ values were 4.3 mM for *A. thaliana*, 4.1 mM for *C. sinensis*, and 3.6 mM for *S. moellendorffii*. However, it is unclear whether anthranilate concentrations would reach 3 to 4 mM or higher *in planta*. *P. vera* had the lowest K_i_ value of 1.8 mM. The other five plant PAT1s have a positively charged Lys in position 264, while the PvPAT1 has a negatively charged Asp in this position, which may account for the low K_i_ value.

### 3-Hydroxyanthranilate acts as an alternative substrate of plant PAT1s

**Figure 5:**
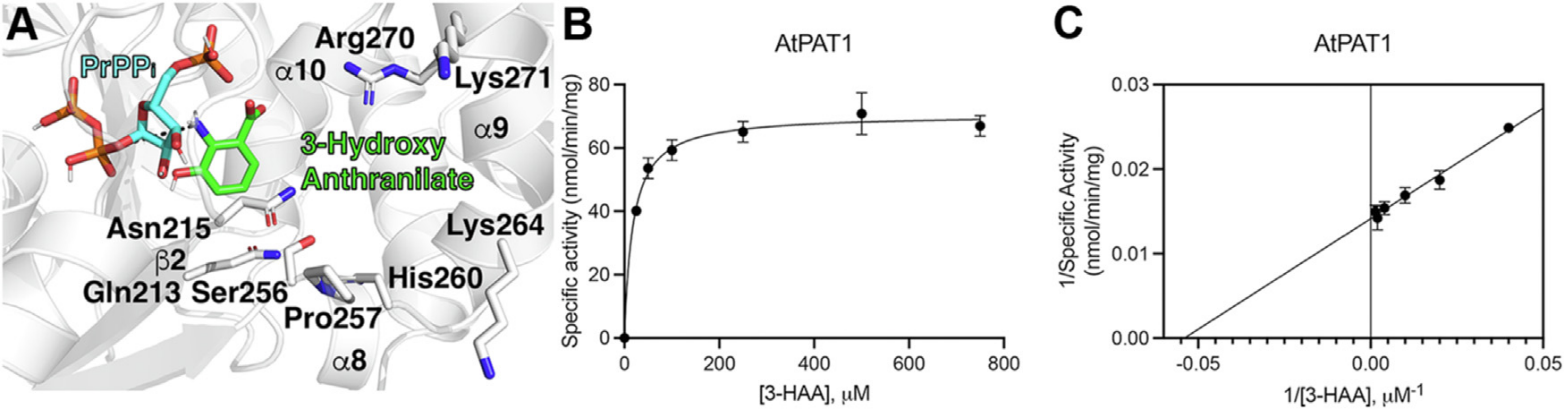
**Plant PAT1s can accept 3-hydroxyanthranilate as a substrate.***A*, molecular docking shows predicted contacts between 3-HAA (*green*) and the AtPAT1 active site. Kinetics data for AtPAT1 are represented on (*B*) Michaelis-Menten and (*C*) Lineweaver-Burke plots across varied 3-HAA concentrations. Error bars represent standard deviations, and where bars are not visible, the standard deviation is too small to visualize. For all points, n = 3.

To probe the substrate specificities of the plant PAT1s, we assayed the enzymes with the mammalian Trp catabolism intermediate 3-hydroxyanthranilate (3-HAA) as a substrate *in vitro*. Very little is known about if and how plants break down amino acids, including Trp, and 3-HAA was selected due to its structural similarity to the cognate substrate anthranilate (35). All six plant PAT1s were able to accept 3-HAA as a substrate (Fig. 5 and Table 2). Interestingly, *A. thaliana* has a similar K_M_ value for 3-HAA (19.63 μM) compared to its K_M_ for anthranilate, while *J. regia* (28.23 μM) and *S. moellendorffii* (22.66 μM) have slightly lower K_M_ values compared to anthranilate. Overall turnover rates with 3-HAA as a substrate ranged from 2.1 to 3.2 min^−1^.

**Table 2:** Steady state kinetics data for plant PAT1s with varied 3-hydroxyanthranilate

PAT1 protein	K_M_ (μM)	k_cat_ (min^−1^)	k_cat_/K_M_ (M^−1^s^−1^)
*Arabidopsis thaliana*	19.63 ± 2.3	2.9 ± 0.07	2470
*Citrus sinensis*	1075 ± 300	2.3 ± 0.3	36.2
*Juglans regia*	28.23 ± 4.2	2.9 ± 0.1	1690
*Physcomitrium patens*	99.71 ± 11.2	3.2 ± 0.1	537
*Pistacia vera*	50.03 ± 5.3	2.1 ± 0.06	711
*Selaginella moellendorffii*	22.66 ± 1.5	3.2 ± 0.05	2360

K_M_ apparent, k_cat_, and k_cat_/K_M_ values reported for 3-HAA concentrations between 0 and 2 mM for *C. sinensis* and between 0 and 0.5 mM for all other enzymes. Values shown are the mean ± standard error for n = 3.

Initially, we saw such low activity for 3-HAA with the *C. sinensis* PAT1 that we did not think that it was able to accept this non-cognate substrate. Upon closer investigation, we were able to calculate Michaelis-Menten kinetics for 3-HAA and show that *C. sinensis* can use it as a substrate, but the catalytic efficiency with 3-HAA (36.2 M^−1^s^−1^) is less than 1% of the activity that the CsPAT1 has with anthranilate, suggesting that the CsPAT1 is highly specific for anthranilate. Similarly, the *P. patens* and *P. vera* PAT1s display 3- and 1.75-fold lower catalytic efficiency with 3-HAA compared to anthranilate, respectively.

Conversely, two PAT1s had a catalytic efficiency with the non-cognate substrate that was the same as or higher than with anthranilate. *J. regia* PAT1 displays a 1.2-fold increased catalytic efficiency with 3-HAA compared to anthranilate, but it is within the standard error of the anthranilate value. The *S. moellendorffii* PAT1 has a 5-fold higher k_cat_/K_M_ with 3-HAA compared to anthranilate, which may mean that there has been little selective pressure for the SmPAT1 to evolve high specificity for anthranilate.

### Identification of residues that contribute to PAT1 activity and promiscuity

To determine the residues responsible for PAT1 substrate promiscuity, we began by comparing active site sequences to identify residues that are shared among 3-HAA-using PAT1s. Two residues that had previously been noted as varying between the *C. sinensis* PAT1, which has high specificity for anthranilate, and other PAT1s were Asn215, which is a Ser in CsPAT1, and Pro257, which is a Thr in CsPAT1. To probe the role of these residues in catalysis and substrate specificity, we introduced the N215S and P257T single mutants and the combined N215S/P256T double mutant into the *A. thaliana* PAT1. If these residues contribute to substrate selectivity, we expected to see reduced activity in the AtPAT1 when the residues from CsPAT1 were introduced. Steady-state kinetics were determined for all three mutants with anthranilate and with 3-HAA (Tables 3 and 4).

**Table 3:** Steady state kinetics data for AtPAT1 mutants with varied anthranilate

Protein	K_M_ (μM)	k_cat_ (min^−1^)	k_cat_/K_M_ (M^−1^s^−1^)	K_i_ (mM)
AtPAT1	20.6 ± 2.0	7.9 ± 0.1	6360	4.3 ± 0.4
AtPAT1 N215S	24.2 ± 3.7	5.2 ± 0.2	3570	>5
AtPAT1 P257T	42.0 ± 2.1	5.8 ± 0.1	2320	4.5 ± 0.5
AtPAT1 N215S/P257T	43.4 ± 6.5	4.5 ± 0.2	1740	>5

K_M_ apparent, k_cat_, and k_cat_/K_M_ values reported for anthranilate concentrations between 0 and 0.75 mM. Values shown are the mean ± standard error for n = 3. Data from Table 1 for the *A. thaliana* PAT1 was included for comparison.

**Table 4:** Steady state kinetics data for AtPAT1 mutants with varied 3-hydroxyanthranilate

Protein	K_M_ (μM)	k_cat_ (min^−1^)	k_cat_/K_M_ (M^−1^s^−1^)
*Arabidopsis thaliana*	19.63 ± 2.3	2.9 ± 0.07	2470
AtPAT1 N215S	65.06 ± 5.5	2.4 ± 0.06	613
AtPAT1 P257T	35.95 ± 6.1	2.9 ± 0.1	1360
AtPAT1 N215S/P257T	60.96 ± 6.4	3.1 ± 0.1	850

K_M_ apparent, k_cat_, and k_cat_/K_M_ values reported for 3-HAA concentrations between 0 and 0.5 mM. Values shown are the mean ± standard error for n = 3. Data from Table 2 for the *A. thaliana* PAT1 was included for comparison.

While the K_M_ value for anthranilate with the AtPAT1 N215S mutant (24.2 μM) was similar to that of the wild-type enzyme (20.6 μM), a reduction in the turnover rate to 5.2 min^−1^ led to an almost 50% reduction in catalytic activity (Table 3). The AtPAT1 P257T and N215S/P257T mutants doubled the K_M_ value for anthranilate (42.0 and 43.4 μM, respectively) and reduced the turnover rate to 5.8 and 4.5 min^−1^, respectively. These data suggest that the Asn215 residue is important for enzyme activity and catalysis, while Pro257 may facilitate anthranilate binding.

When assayed with the alternative substrate 3HAA, the AtPAT1 N215S mutant reduced the catalytic efficiency 4-fold compared to the wild-type enzyme (613 *versus* 2470 M^−1^s^−1^; Table 4). This reduction was in part due to the 3.3-fold higher K_M_ value of the mutant (65 μM) and 17% reduction in k_cat_ (2.4 min^−1^). While the K_M_ and k_cat_ values were also reduced for the P257T mutant, the turnover rate (2.9 min^−1^) was the same as the wild-type value, which meant that the catalytic efficiency was only reduced by 1.8-fold (1360 M^−1^s^−1^) compared to the wild-type enzyme. We did not see an additive effect in the N215S/P257T double mutant. Instead, the enzyme had a further increased turnover rate (3.1 min^−1^) and a K_M_ value (61 μM) that was closest to that of the N215S single mutant. Taken together, these mutagenesis experiments highlight the role of the Asn215 residue in substrate promiscuity.

### Enzyme-level regulation of PAT1

Although PAT1s lack an allosteric site, we questioned whether these enzymes could be regulated by amino acid effectors. While there is evidence of a bacterial PAT1 from the Gram-positive *Brevibacterium flavum* (now *Corynebacterium glutamicum*) being feedback-inhibited by Trp with an IC_50_ value of 0.15 μM, a regulatory mechanism for eukaryotic PAT1s has not yet been reported (22). Furthermore, the *C. glutamicum* PAT1 is specific for *L*-Trp and is not inhibited by Tyr, Phe, His, or *D*-Trp. In our inhibition assays, we included *L*-Trp, as well as the other two aromatic amino acids *L*-Tyr and *L*-Phe. The broader family of phosphoribosyltransferases catalyze the first step in His biosynthesis, and the *A. thaliana* ATP-PRT1 and ATP-PRT2 are feedback inhibited by *L*-His with IC_50_ values of 40 and 320 μM *L*-His, respectively (36). Therefore, we also included *L*-His in our inhibition assays.

**Figure 6:**
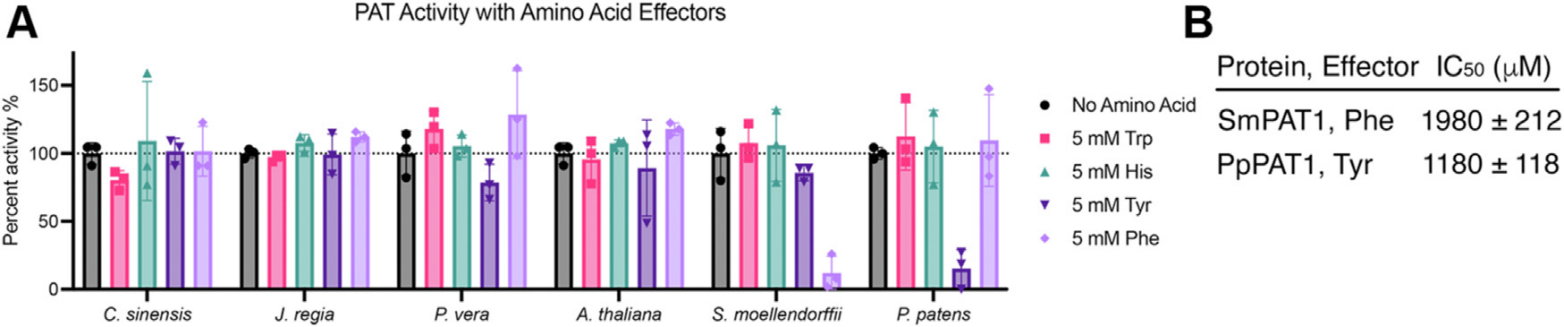
**Screening amino acid effectors of plant PAT1s.***A*, plant PAT1s were each assayed with 5 mM Trp, His, Tyr, or Phe, and activity was normalized to a no amino acid control for each enzyme. Error bars represent standard deviations for n = 3. *B*, inhibition kinetics were determined for SmPAT1 with Phe and PpPAT1 with Tyr by holding anthranilate and PR-PP_i_ constant while varying the concentration of amino acid from 0 to 5 mM. Values shown are the mean ± standard error for n = 3.

None of the six enzymes that we tested were modulated by *L*-Trp or *L*-His, and we did not see significant differences in activity for the CsPAT1, JrPAT1, PvPAT1, or AtPAT1 with 5 mM amino acids added to the reaction (Fig. 6). The SmPAT1 was inhibited by 5 mM Phe (12% activity compared to the “no amino acid” control), while PpPAT1 was inhibited by 5 mM Tyr (15% activity compared to the “no amino acid” control). When the concentration of the amino acid was varied, we calculated an IC_50_ value of 1980 μM for Phe with SmPAT1 and 1180 μM for Tyr with PpPAT1 (Fig. 6). While we are uncertain of the physiological relevance of these IC_50_ values, this data suggests that if Phe levels were high in *S. moellendorffii* (or if Tyr levels were high in *P. patens*), then PAT1 activity would be attenuated.

## Discussion

Since the initial discovery of the *TRP1* (*PAT1*) gene in *Arabidopsis*, Trp biosynthetic enzymes have remained to be revisited and investigated in the post-genomic era (13). As a single-copy gene in plants, all fixed carbon flux to indole and Trp for protein synthesis, specialized metabolism, and auxin hormone biosynthesis proceeds through PAT1. Here we report the first comprehensive investigation of PAT1 across diverse plants to identify variations in activity, efficiency, specificity, and enzyme-level regulation.

Biochemical investigations of metabolic enzymes usually kinetically characterize one or a few enzymes, but taking a broader approach of studying six variable PAT1 enzymes from plants has enabled us to examine the evolution of combinations of active site residues. Using a combination of structural modeling, active site comparisons, and functional assays, we show that PAT1s across plants as diverse as *S. moellendorffii* and *C. sinensis* have evolved varying K_M_ values and catalytic efficiencies (Table 1). Of the plant PAT1 enzymes we tested, *P. vera* PAT1 had the lowest K_i_ value at 1.8 mM. The catalytic cycle and binding mechanism, elucidated by structural studies of PAT1 from the tuberculosis-causing *M. tuberculosis*, could explain why other PAT1s also exhibit substrate inhibition, since binding of multiple anthranilate molecules could prevent folding, trapping pyrophosphate and disrupting catalysis (32). Interestingly, of the PAT1s that we assayed, the *P. vera* PAT1 is the only plant PAT1 that has an Asp substituted for a Lys at the position that corresponds to 264 in *A. thaliana*, which implicates that this residue may be responsible for this low K_i_ value (Fig. 2).

Based on homology to the archaeal *Sulfolobus solfataricus* PAT1 (SsPAT1), we can infer the importance of several residues in anthranilate binding in plant PAT1s (30). Using an X-ray crystal structure and functional assays for the wild-type and mutant SsPAT1, Arg164 (which corresponds to Arg270 in *A. thaliana*) was identified as the main determinant for anthranilate recognition and works by coordinating both the carboxyl and amine of anthranilate. Mutating the SsPAT1 His154 (His 260 in AtPAT1) or His107 (Gln213 in AtPAT1) to an Ala had little effect on binding or catalysis, implying that these residues were dispensable. In SsPAT1, the R164A mutant had a 700-fold higher K_M_ value and a 7-fold decrease in k_cat_, which the authors say implicates this residue in binding but not catalysis. Marino and colleagues suggest that the SsPAT1 Asn109 (Asn215 in AtPAT1) assists Arg164 in binding and orienting anthranilate. We found that the AtPAT1 N215S mutant, when assayed with anthranilate, has a 1.2-fold higher K_M_ value (20.6 μM in wild type to 24.2 μM in the N215S mutant), and the k_cat_ decreased from 7.9 min^−1^ for wild type to 5.2 min^−1^ for the mutant (a 34% decrease) (Table 3). This active site Ser residue is also found in PAT1s of grasses (*Triticum aestivum* (wheat), *Oryza* spp. (rice), *Z. mays* (maize), *Setaria* spp.), *Solanum tuberosum* (potato), *Trifolium pratense* (red clover), *Medicago truncatula* (alfalfa), *Prunus avium* (sweet cherry), and *Prunus dulcis* (almond) (File S1).

In our experiments, we noticed that Asn215 is conserved across plant PAT1s that have a high catalytic efficiency with 3-HAA (*A. thaliana*, *J. regia*, and *S. moellendorffii*) and is a Ser in PAT1s that have a low catalytic efficiency with 3-HAA (*C. sinensis* and *P. patens*) (Table 2; Figs. 2 and 5). The AtPAT1 N215S mutant has a 3.3-fold higher K_M_ value and only a 20% reduction in k_cat_, suggesting that this residue is important for binding 3-HAA but not for catalysis (Table 4). Based on docking results, the side chain of either Asn or Ser in position 215 may be positioned to form hydrogen bond interactions with the hydroxyl of 3-HAA (Fig. 2). Because having an Asn in this position leads to a lower K_M_ value with 3-HAA, the slightly larger polar residue, Asn, may more readily form hydrogen bond interactions with 3-HAA compared to the smaller polar amino acid, Ser.

Of the 82 PAT1s that we investigated, only one, the *C. sinensis* PAT1, has a Thr in the position corresponding to 257 in *A. thaliana* PAT1. Introducing this Thr into AtPAT1 led to a 2-fold increase in K_M_ for anthranilate and a modest 27% decrease in k_cat_, suggesting that this residue is responsible for anthranilate binding (Table 3). Because Pro is a nonpolar and conformationally rigid amino acid, replacing it with a polar Thr may allow for hydrogen bond interactions with anthranilate.

Others have shown that the PAT1 found in *M*. *tuberculosis* can accept a variety of substrates, including analogs of anthranilate that have been fluorinated or methylated on the aromatic ring (31). To our knowledge, no one has reported 3-hydroxyanthranilate activity for a PAT1 enzyme. Because a Trp catabolism pathway has not been identified in plants, the physiological relevance of this reaction is unknown. We were surprised to find that the SmPAT1 has a 5-fold higher catalytic efficiency with 3-HAA compared to anthranilate, primarily due to the 8-fold reduction in K_M_ with 3-HAA (Tables 1 and 2). On the contrary, 3-HAA activity was almost undetectable with the CsPAT1, suggesting that this enzyme is highly specific for anthranilate. Regardless of the physiological relevance, determining plant PAT1 activity with 3-HAA was a useful proxy for determining substrate specificity and revealed a range of promiscuity across plant PAT1s.

Given the high degree of active site conservation among plant PAT1s compared to the PAT1 from *Corynebacterium* spp. that is inhibited by Trp, we hypothesized that plant PAT1s may also be inhibited by Trp. On the contrary, we did not detect Trp inhibition for any of the six plant PAT1s that we assayed (Fig. 6). The PAT1s from *A. thaliana*, *C. sinensis*, *J. regia*, and *P. vera* were insensitive to Trp, Tyr, Phe, and His, while the PAT1 from *S. moellendorffii* and *P. patens* were inhibited by Phe and Tyr, respectively (Fig. 6). Because this inhibition is not shared across the plant PAT1s that we assayed, we hypothesize that the ancestor of plant PAT1s may have been sensitive to amino acid effectors and that modern plants are able to regulate the anthranilate branch point effectively and thus are insensitive to amino acid regulation. We were surprised that the two PAT1s from non-seed plants exhibited amino acid sensitivity, while the PAT1s from angiosperms were insensitive to regulation by Tyr, Phe, Trp, and His. Future experiments are needed to reveal the molecular evolution of amino acid sensitivity in non-seed *versus* seed plants.

Taken together, our findings have led us to hypothesize that because species in the Citrus family (Rutaceae) synthesize anthranilate-derived specialized metabolites like acridone alkaloids, PAT1 has evolved to compete with anthranilate-using enzymes in specialized metabolism. Arabidopsis and other Brassicaceae family members are not known to produce *N*- and *O*-methyl anthranilate, and the selective pressure for high substrate specificity may be reduced. This competition for anthranilate may have led to the high substrate selectivity that is exhibited by CsPAT1. Because Trp is a precursor to a number of industrially valuable molecules, such as inks, dyes, edible essences, and plant growth hormones, using the CsPAT1 for metabolic engineering would be ideal because of its high catalytic efficiency, high fidelity, and insensitivity to feedback regulation (37).

In summary, we report the first biochemical investigation of plant PAT1s. We examined the activity, specificity, and regulation of six enzymes, which informs our understanding of the molecular evolution of Trp biosynthesis in plants.

## Experimental procedures

### Phylogenetic analysis and sequence comparisons of PAT1 proteins

Amino acid sequences for the PAT1 proteins from *A. thaliana* (NP_197300.1), *C. sinensis* (XP_006476804.2), *J. regia* (XP_035550096.1), *P. vera* (XP_031287690.1), *P. patens* (XP_024400584.1), *S. moellendorffii* (XP_024516100.1), and *Corynebacterium* spp. (WP_004567950.1) were obtained from NCBI. Full-length amino acid sequences were aligned using MUSCLE with UPGMA clustering, and a maximum likelihood phylogenetic tree was produced using 1000 bootstrap replicates with MEGA X v10.1.8 *via* the Whelan and Goldman model and a discrete Gamma distribution to model evolutionary rate differences among sites (38).

### Docking and molecular visualization of PAT1 proteins

An AI-generated structure of the *A. thaliana* PAT1 (Uniprot A0A178U8D1) was downloaded from the AlphaFold Protein Structure Database (alphafold.ebi.ac.uk). Phosphoribosyl pyrophosphate (PR-PP_i_) was docked into the active site using AutoDock Vina (ver. 1.5.6) with grid box dimensions of 40 × 40 × 40 Å and the exhaustiveness set to 8 (39, 40). The PR-PP_i_-bound model of the *Arabidopsis* PAT1 was used to perform docking with anthranilate and 3-hydroxyanthranilate using the same grid box dimensions. Docking results were visualized using PyMOL (version 2.5.4).

Putative active site amino acids that were within 5 Å of docked anthranilate were noted for their potential role in catalysis. Residues fell within positions 213 to 215 and 256 to 271 in *A. thaliana* PAT1. To compare PAT1 active site residues across plants, the AtPAT1 amino acid sequence (AAB02913.1) was used as a query in an NCBI protein-protein BLAST search against the Reference Proteins database. Amino acid sequences across 82 diverse plant lineages were selected for comparison and aligned using Clustal Omega. Residue conservation and variability was graphically visualized using WebLogo 3.7.12 (41).

### PAT1 protein expression and purification

The PAT1 amino acid sequences were used for codon-optimized gene synthesis and cloning into the pET28a expression vector (Twist Biosciences). A 5 ml LB culture of *E. coli* BL21 (DE3) harboring the PAT1-containing pET28a plasmid was grown overnight at 37 °C and 225 rpm. The following day, 1 ml of this culture was used to inoculate a 50 ml LB culture supplemented with kanamycin, which was then grown for approximately 16 h at 37 °C and 225 rpm. One-liter cultures of Terrific broth supplemented with kanamycin were inoculated with 25 ml of the 50 ml overnight culture, and the cells were grown at 37 °C and 225 rpm until *A*_600nm_ was between 0.6 and 0.8. At this point, protein expression was induced using a final concentration of 1 mM IPTG (isopropyl ß-D-1-thiogalactopyranoside). After an approximately 16-h incubation at 16 °C, cells were pelleted by centrifugation (5000*g*; 15 min) and resuspended in lysis buffer (50 mM Tris (pH 8.0), 500 mM NaCl, 20 mM imidazole, 10% glycerol, and 1% Tween-20). Following sonication, cell debris was cleared by centrifugation (13,000*g*; 60 min) and the resulting lysate was passed over a Ni^2+^-nitrilotriacetic acid (ThermoScientific) column equilibrated in one column volume of the lysis buffer. The column was washed (50 mM Tris (pH 8.0), 500 mM NaCl, 20 mM imidazole, and 10% glycerol), and bound His-tagged protein was eluted (50 mM Tris (pH 8.0), 500 mM NaCl, 250 mM imidazole, and 10% glycerol). Protein aliquots were stored at −80 °C. Protein concentration was determined by the Bradford method (Bio-Rad) with bovine serum albumin as standard.

### Site-directed mutagenesis cloning

The codon-optimized *AtPAT1* gene in the pET28a plasmid was used as a template for mutagenesis PCR, and oligonucleotides were designed using PrimerX (https://www.bioinformatics.org/primerx/) (Table S1). Mutations were introduced using the QuikChange site-directed mutagenesis method (Agilent), and the successful introduction of each mutation was determined by Sanger sequencing (Azenta). Each construct was transformed into *E. coli* BL21 (DE3) competent cells (New England Biolabs) for heterologous protein expression and purification. Site-directed mutants of PAT1 were expressed and purified using the same methods as wild-type proteins.

### Steady-state kinetic analysis of wild-type and mutant PAT1 enzymes

The transfer of a phosphoribosyl group onto anthranilate was detected by coupling pyrophosphate release from phosphoribosyl pyrophosphate to NADH oxidation at 340 nm using the pyrophosphate reagent, which fructose-6-phosphate kinase (pyrophosphate-dependent), aldolase, triosephosphate isomerase, and α-glycerophosphate dehydrogenase (Millipore Sigma). Following the manufacturer’s instructions, one vial of pyrophosphate reagent was resuspended in 4 ml of milliQ water with a final buffer concentration of 45 mM imidazole HCl (pH 7.4). Phosphoribosyl pyrophosphate (1 mM; Cayman Chemical) was held constant, and the PAT1 enzymes were assayed with anthranilate (Millipore Sigma) or 3-hydroxyanthranilate (0–5 mM; Cayman Chemical) in a final volume of 100 μl in a 96-well plate. Assays were performed at 30 °C and were initiated by the addition of 5 μg of enzyme. Two moles of NADH were oxidized to NAD^+^ for everyone 1 mol of pyrophosphate was consumed, which was taken into account in our specific activity calculations. Initial velocity data were fit to the Michaelis-Menten or substrate inhibition equation using GraphPad Prism (version 9.5.1).

### Amino acid inhibition assays

Amino acid inhibition of PAT1 proteins was screened using an EnzChek Pyrophosphate Assay Kit (ThermoFisher), which couples pyrophosphate release to an inorganic pyrophosphatase and a purine nucleoside phosphorylase (PNP). Reactions were buffered using the provided reaction buffer (50 mM Tris HCl, pH 7.5, 1 mM MgCl_2_, and 0.1 mM sodium azide) and contained 10 μg of the PAT1 enzyme, 0.5 mM anthranilate, 1 mM phosphoribosyl pyrophosphate, and 5 mM of either Trp, His, Phe, or Tyr amino acids (Millipore Sigma) in a final volume of 100 μl. Assays were performed at 30 °C at 360 nm to detect the conversion of 2-amino-6-mercapto-7-methylpurine ribonucleoside by PNP and were initiated by the addition of PAT1 substrates, per the manufacturer’s instructions.

## Data availability

All data are contained within the manuscript.

## Supporting information

This article contains supporting information.

## Conflict of interest

The authors declare that they have no conflicts of interest with the contents of this article.
