# Supplementary material for: Biochemical investigation of the tryptophan biosynthetic enzyme anthranilate phosphoribosyltransferase in plants
Source: J Biol Chem. 2023 Aug 31;299(10):105197. doi: 10.1016/j.jbc.2023.105197 (PMC10520873; doi:10.1016/j.jbc.2023.105197)
Supplement: Supporting information [file mmc1.docx]

**Title**: Biochemical investigation of the tryptophan biosynthetic enzyme anthranilate phosphoribosyltransferase in plants

**Authors**: Miriam Li^1^, Hisham Tadfie^1^, Cameron G. Darnell^1^, and Cynthia K. Holland^1^

**Author affiliations**:

^1^Department of Biology, Williams College, Williamstown, MA 01267, USA.

**Corresponding author information:** Cynthia K. Holland

Tel: +1-413-597-2746, Email: [ckh2@williams.edu](mailto:ckh2@williams.edu)

**Supporting Information**

**Table S1. Site-directed mutagenesis primers for AtPAT1.**

**File S1. Active site residues across 81 plant PAT1 proteins.**

**Table S1. Site-directed mutagenesis primers for AtPAT1.**

| **Primer** | **Sequence** |
| --- | --- |
| N215S-F | 5'-GTTGCCAAACAGGGCAGCCGCTCCAGCTCGTCG-3' |
| N215S-R | 5'-CGACGAGCTGGAGCGGCTGCCCTGTTTGGCAAC-3' |
| P257T-F | 5'GCGGCATTGGCTTTATGATGAGCACCATGTATCATCCTGCGATGAAAATC-3' |
| P257T-R | 5'GATTTTCATCGCAGGATGATACATGGTGCTCATCATAAAGCCAATGCCGC-3' |

**File S1. Active site residues across 81 plant PAT1 proteins.**

>XP_017255324.1 QGN--SPKYHPAMKIVAPVRKK 260

>XP_034708216.1 QGN--SPKYHGAMKIFAPVRKK 255

>XP_027074435.1 QGN--SPNYHPAMKIVAPVRKK 261

>XP_044376826.1 QGS--SANYHPAMKIVRPVRKK 251

>NP_001130955.2 QGS--SANYHPAMKIVRPVRKK 250

>XP_004985893.1 QGS--SANYHPAMKIVRPVRKN 252

>XP_034575219.1 QGS--SANYHPAMKIVRPVRKN 252

>XP_040378099.1 QGS--SANYHPAMKIVKPVRKK 252

>XP_015630986.1 QGS--SANYHPAMKIVKPVRKK 254

>XP_052146826.1 QGS--SANYHPAMKIVKPVRKK 254

>XP_031476324.1 QGN--GPEYHPAMKIVGPVRKK 253

>XP_020249535.1 QGN--SPIYHPAMKIVSPVRKK 267

>XP_021857819.1 QGN--SPTYHPAMKVVRPIRKQ 266

>XP_043635834.1 QGN--SPIYHPAMQIVRPVRKQ 256

>XP_023765044.1 QGN--SPIYHPAMQIVRPVRKQ 256

>XP_021981996.1 QGN--SPVYHPAMQIVRPVRKQ 240

>XP_027168161.1 QGN--SPIYHPAMKIVRPIRKK 259

>XP_027123105.1 QGN--SPIYHPAMKIVRPIRKK 259

>XP_019175440.1 QGN--SPIYHPAMKIVSPVRKK 260

>XP_031090695.1 QGN--SPIYHPAMKIVSPVRKK 260

>XP_009762500.1 QGN--APVYHPAMKIVRPIRKK 251

>XP_055831681.1 QGN--SPIYHPAMKIVRPIRKK 254

>XP_049408844.1 QGN--SPIYHPAMKIVRPIRKK 253

>XP_006347447.1 QGS--SPIYHPAMKIVRPIRKK 253

>XP_015077051.1 QGN--SPIYHPAMKIVRPIRKK 253

>XP_022889880.1 QGN--APMYHPAMKIVRPIRKK 265

>XP_042039455.1 QGN--SPIYHPAMKIVKPVRSK 250

>XP_051139148.1 QGN--SPIYHPAMQIVRPVRKK 260

>XP_007138060.1 QGN--SPKYHPSMKIVRPIRKK 252

>XP_020231197.1 QGN--SPKYHQAMKIVRPVRKK 247

>XP_027334801.1 QGN--SPKYHPAMKIVRPVRKK 256

>XP_004501916.1 QGN--SPMYHPAMNIVRPVRRK 286

>XP_045821638.1 QGS--SPKYHPAMKIVRPVRKQ 255

>XP_003601245.1 QGS--SPKYHPAMKIVRPVRKK 247

>XP_006476804.2 QGS--STKYHPAMKFVRPVRKK 266

>XP_013683125.2 QGN--SPMYHPAMKIVGPVRKK 288

>XP_009121033.1 QGN--SPMYHPAMKIVGPVRKK 284

>XP_010420535.1 QGN--SPMYHPAMKIVGPVRKK 296

>XP_010454019.1 QGN--SPMYHPAMKIVGPVRKK 297

>NP_197300.1 QGN--SPMYHPAMKIVGPVRKK 299

>XP_006287763.1 QGN--SPMYHPAMKIVGPVRKK 296

>XP_026387238.1 QGN--SPIYHPAMKIVGPVRKK 256

>XP_042497171.1 QGN--APKYHPAMKIVGPVRKK 254

>XP_043707651.1 QGN--APKYHPAMKIVGPVRKK 255

>XP_008374423.1 QGN--APKYHPAMHIIRPVRKK 257

>XP_021826008.1 QGS--APKYHPAMNVVRPVRKK 257

>XP_034216600.1 QGS--APKYHPAMNVVRPVRKK 257

>XP_011464025.1 QGN--APKYHSAMRIVRPVRKK 256

>XP_050387924.1 QGN--APKYHPAMRVIRPVRKK 253

>XP_040364131.1 QGN--APKYHPAMNVVRPVRKK 258

>XP_024173045.1 QGN--APKYHPAMNVVRPVRKK 258

>XP_024163269.1 QGN--APKYHPAMNVVRLVRKK 255

>XP_031403552.1 QGN--GPIYHPAMKIVSPVRKK 267

>XP_030458001.1 QGN--APKYHPAMKIVSPVRKK 259

>XP_010027548.1 QGN--APKYHPAMKIVSPVRKK 258

>XP_016728110.1 QGN--APKYHPAMKIVSGVRKK 262

>XP_012440436.1 QGN--APKYHPAMKIVSGVRKK 262

>XP_022736987.1 QGN--APKYHPAMKIVSPVRKK 269

>XP_021292004.1 QGN--SPKYHPAMKIVSPVRKK 269

>XP_022137235.1 QGN--APKYHPAMKIVSPVRKK 277

>XP_022137236.1 QGN--APKYHPAMKIVSPVRKK 277

>XP_004152482.1 QGN--APKYHPAMKIVSPVRKK 271

>XP_023519343.1 QGN--SPKYHPAMKIVGPVRKK 272

>XP_023000687.1 QGN--SPKYHPAMKIVSPVRKK 272

>XP_011001513.1 QGN--SPKYHPAMKIVSPVRKK 274

>XP_024437667.1 QGN--SPKYHPAMKIVSPVRKK 269

>XP_034909266.1 QGN--SPKYHPAMKIVGPVRKK 269

>XP_034909265.1 QGN--SPKYHPAMKIVGPVRKK 341

>XP_028059754.1 QGN--APIYHPAMKIVSPVRKK 254

>XP_012080040.1 QGN--SPKYHPAMKIVSPVRKK 267

>XP_048228972.1 QGN--SPKYHPAMKIVSAVRKK 277

>XP_021615970.1 QGN--SPKYHPAMKIVSPVRKK 276

>XP_035550096.1 QGN--APKYHPAMKIVSPVRKT 256

>XP_050260716.1 QGN--APRYHPAMKIVSPVRKK 255

>XP_023919375.1 QGN--SPRYHPAMKIVSPVRKK 256

>XP_030930448.1 QGN--SPRYHPAMKIVSPVRKK 256

>XP_010110365.2 QGN--APIYHPAMKIVRPVRKK 257

>XP_015882552.2 QGN--APIYHPAMKIVRPIRKK 259

>XP_024400584.1 HGS--APTYHPAMKVVAPVRRS 235

>XP_031287690.1 HGN--APRYHPAMDIVRPVRKR 237

>XP_024516100.1 HGN--APKFHPAMKVVGPVRKS 213

>PTQ43022.1 HGS--AANYHPAMKVVAPVRKA 233
